# First High-Density Linkage Map and Quantitative Trait Loci for Disease Resistance in Striped Catfish *Pangasianodon hypophthalmus*

**DOI:** 10.3390/ijms27020784

**Published:** 2026-01-13

**Authors:** Nguyen Thanh Vu, Tran Huu Phuc, Tran Thi Mai Huong, Nguyen Hong Nguyen

**Affiliations:** 1 Centre for Bioinnovation, University of the Sunshine Coast, Locked Bag 4, Maroochydore DC, QLD 4558, Australia; thimaihuong.tran@research.usc.edu.au; 2 Research Institute for Aquaculture No.2, 121 Nguyen Dinh Chieu Street, Ho Chi Minh City 700000, Vietnam; tranhuuphuc30@gmail.com; 3 Tropical Futures Institute, James Cook University, 149 Sims Drive, Singapore 387380, Singapore; 4 School of Science, Engineering and Technology, University of the Sunshine Coast, Locked Bag 4, Maroochydore DC, QLD 4558, Australia

**Keywords:** linkage map, QTL, disease resistance, candidate genes, molecular breeding, genetic improvement

## Abstract

While striped catfish (*Pangasianodon hypophthalmus*) is an economically important aquaculture species, its genomic resources remain limited. To date, linkage maps, QTL (quantitative trait loci) analyses, and the identification of candidate genes associated with disease resistance traits are very limited. Therefore, the present study aimed to construct a high-density linkage map and identify candidate genes for this species. Our analysis was conducted on a pedigree population consisting of 560 individuals (490 offspring and 70 parents for 40 families), whose genomes were analyzed using a genotyping-by-sequencing platform. After stringent filtering, 9882 high-quality SNPs were retained for linkage analysis. Linkage analysis placed 8786 markers onto 30 linkage groups (LGs), with an average density of 0.43 SNPs per cM. Recombination rates varied across the 30 linkage groups (LGs), averaging of 3.6 cM/Mb in males, 6.7 cM/Mb in females, and 5.1 cM/Mb when sex-averaged. Using the linkage map, our QTL analysis identified three significant QTLs for disease resistance to *Edwardsiella ictaluri*, the causative agent of Bacillary Necrosis of Pangasius (BNP). The QTLs were located on LG1, LG9 and LG29, and their peak markers explained 17.03% of the phenotypic variance. An LD-based interval of approximately ±25 kb surrounding the QTL peak was identified as the putative candidate region. However, subsequent genome-wide association analysis did not identify significant SNP effects within these regions, suggesting that the QTLs may represent polygenic or small-effect loci that are detectable only in linkage-based analyses. In summary, this study presents the first high-density SNP-based linkage map for striped catfish and reports significant QTL and associated candidate genes related to disease resistance and growth traits. These findings provide valuable insights into the genetic architecture of economically important traits in *P. hypophthalmus*. Nevertheless, further validation in independent populations is required before incorporating these markers into selective breeding programs.

## 1. Introduction

Striped catfish (*Pangasianodon hypophthalmus*) is an important aquaculture species in many countries, especially Vietnam, where annual production reached 1.7 million tons in 2024, valued at 2.0 billion US dollars [1]. However, genomic resources for this species are still limited, despite recent efforts to establish draft genome assemblies [2,3,4,5,6]. Some studies have also attempted to use genome sequences or genome-wide markers to predict phenotypes in *P. hypophthalmus*, achieving moderate to high levels of accuracy (0.51–0.75), for instance, in resistance to Bacillary Necrosis of Pangasius (BNP) caused by *E. ictaluri* [7,8,9] or body weight at tagging [10]. Nevertheless, there are still no published studies or available information on linkage maps for this species. In contrast, several high-density genetic maps have been developed for a range of aquaculture species, including fish [11], crustaceans [12], and molluscs [13].

With the decreasing costs of next-generation sequencing and high-throughput platforms, it is now possible to construct high-density linkage maps to overcome the limitations of earlier versions. High-quality DNA markers such as single-nucleotide polymorphisms (SNPs) can enhance both the density and resolution of linkage maps, improving them to a scale of 1–2 cM or even smaller. Furthermore, these markers facilitate QTL analysis, enabling the detection of genomic regions and candidate genes associated with quantitative complex traits, such as growth characters in Wild dusky kob, *Argyrosomus japonicus* [14], resistance to environmental stressors like salinity in Nile tilapia *Oreochromis niloticus* [15,16], *Vibrio anguillarum* disease resistance in flatfish [17], or vibriosis resistance in tongue sole *Cynoglossus semilaevis* [18].

Furthermore, linkage maps and QTL analysis offer additional benefits. For instance, they help verify whether the reference genome order aligns with genetic linkage groups and can identify potential chromosomal rearrangements, such as inversions, translocations, or mis-assemblies [19,20]. They also allow for the measurement of recombination rate variations across chromosomes and sexes [21]. A high-density linkage map is a cornerstone for trait dissection and breeding as it structures markers into recombining units for robust QTL mapping, anchors and validates genome assemblies, delineates haplotype blocks for imputation, and improves the interpretability and transferability of genomic selection [22,23], which are important to striped catfish breeding.

Practically, fine-mapping of significant QTLs and loci supports the potential application of marker- or gene-assisted selection (MAS) in genetic enhancement programs. A classic example in aquaculture is the unusually large proportion of the genetic variation explained by a QTL (*nae1* gene) involved with infectious pancreatic necrosis (IPN) disease in Atlantic salmon [24]. Recent studies also demonstrated that marker-assisted selection for a QTL (involving two genes: *IRF2* and *Viperin*) associated with survival to OsHV-1 mortality events increased genetic gain for survival in Pacific oyster [25]. Another report that used two sex-linked markers to establish a marker-assisted sex-control technique for the production of mono sex (only male) population for aquaculture and breeding of WW individuals for study of sex determination in blue tilapia [26]. There are also other examples of the integration of MAS for resistance to nervous necrosis virus (NNV) and iridovirus, as well as high omega-3 content in Asian seabass [27] or for commercial traits of aquaculture species [28].

To explore the prospects of marker-assisted selection for striped catfish, we conducted linkage and QTL mapping for economically important traits, particularly disease resistance to *Edwardsiella ictalurid*, the causative agent of Bacillary Necrosis of Pangasius (BNP) [29]. This is crucial because BNP disease caused by this pathogen has resulted in significant economic losses for the sector, estimated at 48 to 94 million US dollars per annum [8].

The ultimate aim of this study is to enhance disease resistance of striped catfish. The specific objectives include the following:

(i) constructing a high-density linkage map using 10k SNPs generated through genotyping-by-sequencing platforms,
(ii) conducting QTL and trait association analyses, and
(iii) identifying potential candidate genes involved in two key traits: resistance time to BNP disease and survival rate.

Our findings are expected to improve our understanding of the genomic architecture of these complex traits and provide fundamental information for designing alternative breeding schemes for future selection in this species.

## 2. Results

### 2.1. Phenotype and Quantitative Genetic Basis

**Figure 1:**
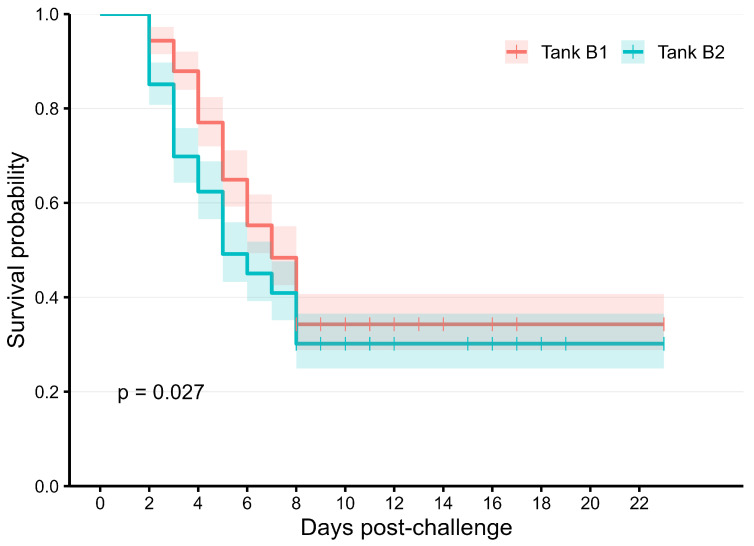
Kaplan–Meier curve of the survival rate in the studied population.

The average tagging weight of the experimental population was approximately 21 g, about 2.5–3.0 months after hatching. The fish were challenged with the *E. ictaluri* pathogen in tanks over a period of 23 days. Mortality began after about 2–3 days, peaked at 7 days, and then generally plateaued for the remainder of the experimental period (Figure 1). The overall survival rate of the studied population was 32% (Table 1). Heritability estimates for both survival status and survival time are moderate (0.46–0.49), and their genetic correlations were high (0.69 ± 0.05).

### 2.2. Sequencing, Variant Calling and Genotype Information

Genome sequence information for 490 offspring, their parents, and the overall population is presented in Table 2. The average number of clean reads ranged from 1.74 to 2.3 million, with approximately 90% successfully mapped to the reference genome of the species.

Genome depth coverage (×) and breadth (%) were calculated relative to the full 732 Mb assembly. Because DArTseq targets only a small fraction of restriction fragments, low genome-wide coverage (0.15×–0.25×) is expected. Importantly, the mean read depth at targeted loci (19.6×–31.6×) is good within the range required for accurate SNP discovery and genotype calling. The slightly higher depth observed in parents reflects their higher-density genotyping and deeper sequencing effort.

The average call rate across all samples (offspring, their parents, and the overall population) was high, ranging from 98.5% to 99.0% (Table 3). The minor allele frequency in the population was about 0.43. The frequency of heterozygosity (i.e., the proportion of samples scored as heterozygous) was moderate, ranging from 0.32 to 0.33. In total, 336,782 SNPs were discovered across this population. After stringent quality control, as described in the Section 4.3, 9882 SNPs (Supplementary Materials Figure S1) remained for downstream analyses for linkage mapping analysis.

### 2.3. Construction of Genetic Linkage Map

**Figure 2:**
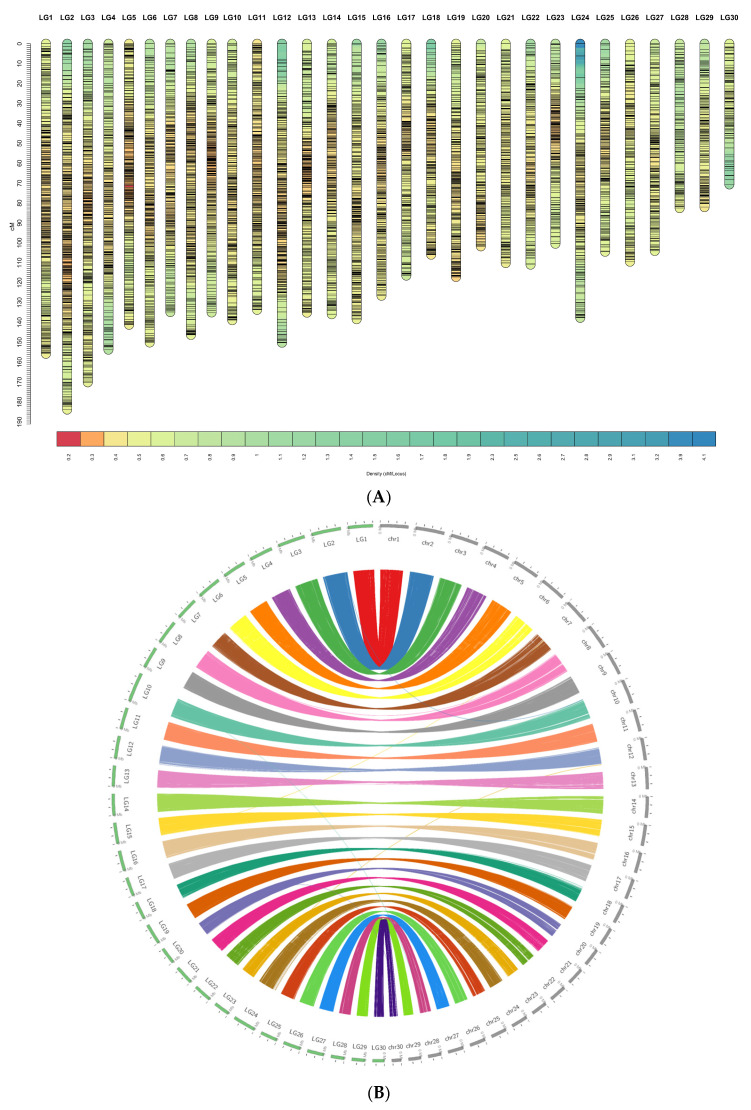
(**A**). Sex averaged linkage map of *Pangasianodon hypophthalmus*. Visualization of marker density along the high-density striped catfish linkage map. Each vertical bar represents a linkage group (LG1–LG30), oriented from 0 cM at the top to increasing genetic distance down the axis (cM). Horizontal segments within each LG show local marker density estimated in sliding windows along the map; colors correspond to marker density according to the scale shown at the bottom (density in markers per cM, as indicated by the legend). Regions with warmer colors indicate higher marker density, whereas cooler colors indicate lower density/sparser coverage. Overall, markers are distributed across all linkage groups, with localized variation in density that may reflect differences in recombination rate or genomic features affecting marker availability. (**B**). Genome anchoring and collinearity between the high-density striped catfish linkage map and the reference genome assembly. Circos plot summarizing the correspondence between linkage groups (LG1–LG30) and reference genome chromosomes (chr1–chr30) using genome-anchored SNP markers. The left arc (green outer track) shows linkage groups ordered around the circle, while the right arc (gray outer track) shows the assembled chromosomes. Tick marks on the outer tracks indicate map/assembly scale (linkage map positions in cM; physical positions along chromosomes was scaled to cM to improve balanced view). Colored ribbons connect each marker (extended in 100 bp) between its position on a linkage group and its physical position on the corresponding chromosome; ribbon colors follow the linkage group identity. The predominance of one-to-one ribbon connections indicates strong concordance between the linkage map and genome assembly, whereas occasional cross-links (thin ribbons connecting non-matching LG–chr pairs) may reflect ambiguous marker placement, repetitive regions, or local assembly/mapping inconsistencies.

By retaining at least 10 SNPs per linkage group, a total of 8786 of 9877 SNPs (88.9%) were mapped across 30 linkage groups (LGs), matching the species’ karyotype (Figure 2A,B). SNP density per chromosome ranged from 110 to 466 markers (mean = 292.87; SD = 80.71) (Table 4). Sex-averaged LG lengths varied from 70.64 to 183.66 cM, with an average of 126.04 cM and a standard deviation of 26.23. An additional 1091 SNP markers could not be assigned to the LGs (less than 10 markers/LG) due to insufficient informative families and individuals. Female-to-Male map length ratio is approximately 1.90 (166.67/89.54), with total lengths of 5000.2 cM and 2686.2 cM for female-biased and male-biased map, respectively. This pattern of heterochiasmy, where females exhibit higher recombination rates than males in this striped catfish dataset, was consistent across all LGs. On average, the female linkage groups measured 166.67 cM in length, compared to 89.54 cM in males. Notably, LG2, LG3 and LG6 exhibited the highest recombination length in both sexes, reaching 111–120 cM in males and 195–252 cM in females. These results suggest sex-specific recombination landscapes that may influence the resolution and accuracy of downstream QTL mapping (Supplementary Materials Figures S2 and S3). This panel of 8786 SNPs was then used for QTL and GWAS.

### 2.4. Synteny Analysis Between Linkage Map and Genome Assembly

Circular synteny plot comparing the genetic linkage map (left semicircle; LG1–LG30) with the chromosome-scale genome assembly (right semicircle; chr1–chr30). Outer tracks show LGs and chromosomes; colored ribbons denote collinear blocks linking homologous regions (Figure 2B). The majority of ribbons form clean one-to-one arcs from each LG to a single chromosome, indicating strong collinearity and confirming assembly contiguity and marker order. A small number of split or tapering ribbons reflect regions where markers span adjacent blocks or where local ordering is less certain. Overall, the plot supports a near chromosome-for-chromosome correspondence between the genetic map and the assembly.

**Figure 3:**
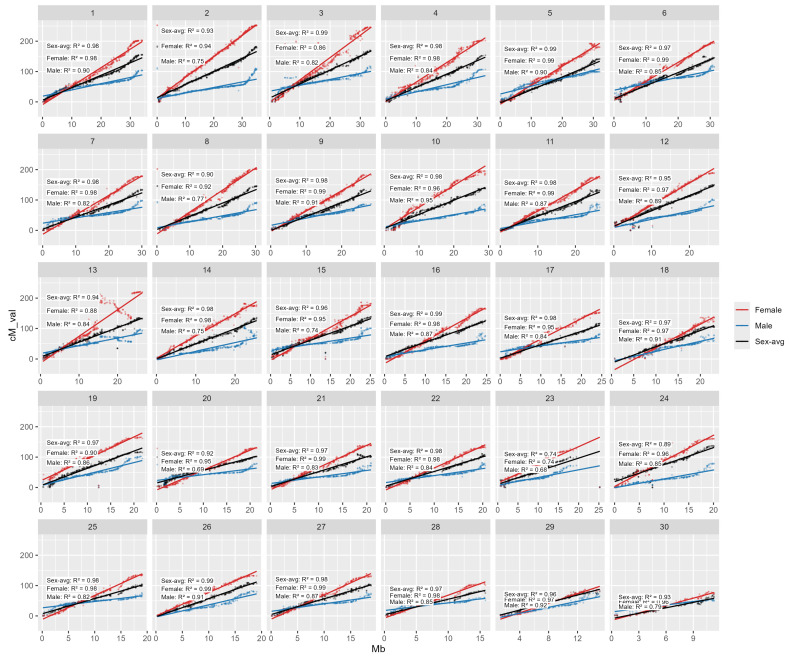
Correlation plot between genetic (male-biased, female-biased and sex-averaged) mapping versus physical mapping position. Scatterplots showing the relationship between physical position (Mb; x-axis) and genetic position (cM; y-axis) for genome-anchored SNP markers across the 30 chromosomes/linkage groups (panels 1–30). For each chromosome, marker positions are plotted and fitted with linear regression lines for the female map (red), male map (blue), and sex-averaged map (black). The R^2^ values shown within each panel quantify the strength of collinearity between genetic and physical order for each map, providing a measure of map–genome concordance. Overall, the consistently high R^2^ values across chromosomes indicate strong agreement between the linkage maps and the reference genome assembly, while chromosomes showing comparatively lower male R^2^ values suggest localized departures from linearity that may reflect sex-specific recombination differences, regions of reduced marker informativeness, or local assembly/mapping uncertainty.

Across the 30 linkage groups, the sex-averaged maps show strong linear concordance with physical position (R^2^ from 0.68 to 0.99) (Figure 3). Female map concordance between physical and assigned LG is consistently greater than that of male which many R^2^ are greater than 0.9 indicating smooth, near-perfect scaling of cM with Mb. Male maps are generally good but weaker on several groups; the lowest is LG8, LG14, LG20, LG21 and LG30 (0.74–0.79), and even lower of LG26 and LG29 (0.68–0.69).

### 2.5. QTL for Disease Traits

**Figure 4:**
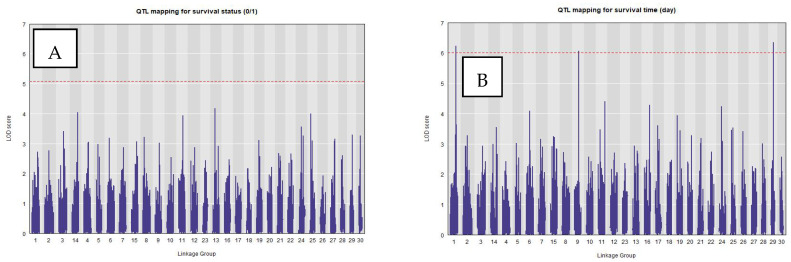
Genome-wide QTL scans for E. ictaluri resistance traits in striped catfish. Genome-wide QTL mapping results from R/qtl2 for two resistance-related traits measured following *Edwardsiella ictaluri* challenge. (**A**) Survival status (0/1) and (**B**) survival time (days) were scanned across 30 linkage groups using 8786 genome-anchored SNP markers. The x-axis shows marker position along concatenated linkage groups (alternating gray shading separates linkage groups), and the y-axis shows the LOD score. The horizontal dashed red line indicates the genome-wide significance threshold, determined by permutation testing. Peaks exceeding (or approaching) this threshold identify QTL regions associated with resistance; in the survival time scan, three prominent peaks correspond to QTLs on LG1, LG9, and LG29.

Permutation tests using the LOCO mixed model indicated genome-wide significance thresholds of approximately LOD of 5.07 and 6.10 for survival status and survival time, respectively. Only two single QTLs on LG1 (LOD = 6.24) and LG29 (LOD = 6.35) for survival time surpassed and one QTL on LG9 (LOD = 6.05) approaching the threshold of 6.10 (Figure 4). Their peak markers (LG1, LG9 and LG29) accounted for 5.7%, 5.5% and 5.8% of the phenotypic variance. Linkage decay analysis using PopLDdecay showed that the flanking region around the peak can be extended by ~25 kb on both sides to capture the local haplotype block associated with these QTLs (Supplementary Materials Figure S4).

Due to potential impact of population structure and relatedness on QTL detection, we additionally compared three mapping approaches: Haley–Knott (KH) regression, a single-kinship linear mixed models (LMMs), and the LOCO mixed model (Supplementary Materials Figures S5 and S6). The Haley–Knott regression detected several significant regions for both survival status and survival time, but this approach assumes constant variance, while linear mixed models (LMMs) are more robust and can account for population structure and kinship. The leave-one-chromosome-out (LOCO) mixed model is an advanced version of LMM that offers more accurate QTL detection than a standard kinship LMM, especially for detecting regions on the same chromosome as the QTL. Our results confirmed that the LOCO approach provided the most conservative and reliable control of polygenic background relatedness, and hence, all subsequent analyses were based on the LOCO model.

### 2.6. QTL Effect Decomposition into Additive and Dominance Components

To quantify the genetic action of each survival-time QTL, we extracted genotype probabilities at the peak positions and partitioned the effects into additive (a) and dominance (d) components using a standard F_2_ parameterization. The decomposition reveals that the three significant QTLs differ strongly in both magnitude and mode of gene action (Table 5).

The QTL on LG1 (116.4 cM) showed a modest additive effect (a = −0.56), equivalent to a 6.1% reduction in survival time per copy of the B allele. The dominance deviation was also negative (d = −0.88), indicating partial dominance of the low-survival allele, with AB individuals displaying lower survival than expected from the midpoint between AA and BB genotypes. In contrast, the LG9 QTL (84.4 cM) displayed a substantial positive additive effect (a = 2.62), representing a 28.7% increase in survival time per B allele. The large positive dominance deviation (d = 3.33) indicates a pattern of overdominance, with heterozygotes exhibiting the highest survival time (12.4 units) compared to either homozygote (6.5–11.7 units). The QTL on LG29 (54.2 cM) exhibited the largest effect, with a strongly negative additive component (a = −4.34), corresponding to a 47.7% reduction in survival per B allele. The dominance deviation was similarly negative (d = −4.59), meaning heterozygotes performed even worse than BB homozygotes. This pattern reflects strong dominance of a deleterious allele, with AA-genotype fish surviving approximately three times longer than AB or BB genotypes.

### 2.7. GWAS of Resistant Traits

Genome-wide association analysis for survival status (Supplementary Materials Figure S7) and survival time (Supplementary Materials Figure S8) did not identify any SNPs exceeding the Bonferroni-adjusted significance threshold (α = 0.05/8786; −log_10_(*p)* = 5.2). Although several loci displayed moderate association peaks (−log_10_(*p)* = 3–4), none surpassed the conservative correction required for multiple testing. The absence of genome-wide significant SNPs is likely attributable to the combined effects of limited sample size (*n* = 490), modest marker density (8786 SNPs), and the polygenic nature of survival traits. These results indicated that no single SNP exerted a large marginal effect detectable under stringent GWAS thresholds, consistent with the QTL mapping results showing that survival is influenced by multiple loci of small-to-moderate effect.

### 2.8. Gene Associated with the Three Peak QTLs

To characterize the genetic content underlying the QTL interval, all annotated features within the corresponding genomic coordinates were extracted from the striped catfish (*P. hypophthalmus*) GFF file (Accession: GCF_009078355.1). This search yielded 17 distinct protein-coding genes after removing structural annotations (CDS, mRNA, ncRNA) and redundant feature entries. The genes varied widely in feature count, reflecting differences in exon–intron structure and transcript representation in the GFF (Supplementary Materials Table S1). Functional annotation revealed a mixture of metabolic, regulatory, and signaling genes, including several with known or plausible roles in immune modulation. Notably, *cd151, chid1, klf6a, inhbaa, slc15a5,* and *srpk2* have documented functions in leukocyte activation, innate immune recognition, cytokine signaling, or antiviral responses, while others (e.g., *pfkpa*, *pus7*, *top2b*) contribute indirectly through transcriptional or metabolic control during immune activation. This curated set of 17 genes was subsequently used as the input gene list for enrichment analyses using g:Profiler pathway searches to investigate overrepresented biological processes and potential mechanistic links to disease resistance using human and zebra fish database.

Functional enrichment analysis using g:Profiler identified several significant GO biological processes (FDR < 0.05, Supplementary Materials Table S2), despite the small number of annotated genes within the QTL interval. The most strongly enriched terms included hemopoiesis (GO:0030097), lymphocyte differentiation (GO:0030098), B-cell differentiation (GO:0030183), lymphocyte activation (GO:0046649) and leukocyte activation (GO:0045321), all of which are central to innate and adaptive immune function. In addition, the term positive regulation of viral genome replication (GO:0045070) was enriched, highlighting a potential antiviral component. Enriched cellular-component terms included the activin/inhibin complex (driven by *inhbaa*) and the Wnt–Frizzled–LRP5/6 signaling complex (driven by *fzd8a*), indicating involvement of TGF-β and Wnt signaling pathways. Together, these enrichment patterns provide strong evidence that the QTL region contains genes involved in immune-cell development, activation, and antiviral defense, consistent with the observed association with survival under disease challenge.

## 3. Discussion

### 3.1. Major Advances of This Study

This study provides the first high-density SNP-based linkage map for striped catfish, an economically important aquaculture species. Using a large, structured pedigree of 40 families and 490 offspring, we generated a linkage map comprising 8786 markers across 30 linkage groups, with an average inter-marker distance of 0.46 cM. Compared with existing genomic studies in this species, which have primarily focused on genome assemblies or genomic prediction, our study fills a critical gap by providing a robust recombination-based framework that supports fine-scale QTL mapping, genome validation, and the development of future marker-assisted breeding strategies. This map also revealed clear heterochiasmy, with female recombination rates nearly two-fold higher than in males (6.76 vs. 3.71 cM/Mb) [30]. Such sex-specific recombination landscapes have been documented in several fish species but were previously unknown for striped catfish. Understanding these differences is important for optimizing QTL detection, interpreting crossover patterns, and designing breeding schemes.

### 3.2. QTL Detection and GWAS

Despite an extensive genome-wide search and the use of an assigned marker panel, we identified three significant QTLs influencing resistance to *Edwardsiella ictaluri*, specifically the trait survival time on LG1, LG9 and LG29. These QTLs exceeded (or were near) the genome-wide significance threshold (LOD ~ 6.1) and explained up to 17.03% of phenotypic variance. Although modest, this effect size is consistent with the expectation that disease resistance in striped catfish is a polygenic trait, as shown in previous quantitative genetic studies [8,31]. Noticeably, the three loci also showed clearly interpretable genotype–phenotype contrasts, indicating distinct genetic architectures across QTLs rather than a single uniform mode of action. For example, genotype means at the QTL peaks suggest heterozygote advantage at LG9 (AB showing the highest survival time), whereas LG29 showed a large genotype shift where AA markedly outperformed AB/BB, consistent with a major susceptibility-associated allele. These patterns imply that non-additive effects (dominance/overdominance) may contribute alongside additive allele substitution, which is biologically plausible for immune traits where balanced or regulated responses can be advantageous under infection [32]. Consequently, these three QTLs are not only statistically significant but also biologically informative because they provide testable hypotheses about how host genotype shapes infection outcomes, and they offer immediate priorities for functional gene nomination and validation within each interval.

Interestingly, no significant associations were detected at the same region using single-step GWAS, suggesting that the QTLs may represent either: (1) a family-specific or pedigree-linked signal, detectable only through linkage-based analyses, or (2) a small-effect locus diluted in a genome-wide association framework. The discrepancy between QTL mapping and GWAS likely reflects differences in statistical power, allele frequency, and linkage disequilibrium structure between family-based and population-based designs. The detected QTLs may represent loci segregating within specific families or small to moderate-effect loci that do not reach genome-wide significance in GWAS. This illustrates the complementary roles of linkage mapping and GWAS in which linkage methods provide power in structured families, whereas GWAS is sensitive to signals consistent across the whole population [33,34,35]. From an applied perspective, this also indicates that validation in independent families and broader populations will be essential before deploying markers for selection, especially for loci showing strong dominance patterns.

### 3.3. Biological Plausible Candidate Genes for Disease Resistance to E. Ictaluri

The finding of biologically plausible functional genes underlying the three significant QTL regions (LG1, LG9, and LG29) represents a central outcome of this study. Although only 17 annotated genes were identified in the QTL interval, enrichment analysis revealed a coherent cluster of immune-related pathways. Several key biological processes involved in hematopoiesis, lymphocyte maturation, immune-cell activation and antiviral signaling [36] were significantly enriched. Genes such as *cd151*, *chid1*, *klf6*, *inhbaa*, and *slc15a5* contributed to most of these terms and have established roles in leukocyte activation [37], innate immunity [37,38], cytokine regulation or immune transcriptional control. The enrichment of the activin/inhibin complex [39] and Wnt–Frizzled signaling [40] further supports regulatory involvement in immune modulation. These findings suggest that the LG1, LG9 and LG29 QTLs harbor an immunologically relevant gene cluster likely contributing to variation in survival following pathogen exposure. Importantly, we interpret these genes as biological plausible candidates rather than confirmed causal drivers: the mapping resolution of linkage QTL and the limited number of genes within the intervals support biological plausibility, but do not establish functional causality. Accordingly, these QTL regions should be viewed as prioritized targets for future research, including fine-mapping to resolve linked variants, evaluation of local LD structure, and functional support from expression profiling (e.g., infection-challenge transcriptomics in relevant tissues) or allele-specific effects. Overall, identification of these QTLs and its candidate genes provides a foundation for understanding quantitative disease resistance in striped catfish and is the first report of a genome-anchored QTL for *E. ictaluri* survival.

### 3.4. Implications for Selective Breeding

Although the QTLs explain only a modest fraction of the variation, its detection across multiple families and clear genotype–phenotype differentiation point to a putative genomic signal. In practical breeding programs, such QTLs when validated, can be integrated through marker-assisted selection (MAS) if a high-impact causal variant is identified, haplotype-assisted breeding to track favorable alleles within families, or genomic selection (GS) informed by LD-defined haplotype blocks. Given the low to moderate effect size, MAS alone is unlikely to drive large genetic gains. However, incorporating validated QTL into multi-trait GS models could improve prediction accuracy for disease resistance, particularly in early selection or low-heritability settings, as previously demonstrated in tilapia, salmon, shrimp and oyster breeding programs [41,42,43,44].

### 3.5. Future Directions

While our results provide valuable insights into the genetic architecture of disease resistance traits in *P. hypophthalmus*, additional validation is required before these markers can be incorporated into selective breeding programs for this species. Fine mapping is necessary to narrow down the QTL regions to smaller genomic intervals with fewer candidate genes [45]. As resources allow, targeted resequencing should be conducted to identify all genetic variants within the fine-mapped regions, followed by variant annotation to predict their potential effects (e.g., synonymous, non-synonymous, stop-gain, regulatory). This information will help prioritize variants most likely to be causative, especially in experimental populations involving affected versus unaffected individuals. Once candidate variants are identified, further functional prediction can be used to prioritize high-impact variants and assess their influence on gene expression, particularly through the identification of cis-eQTLs that affect candidate genes. Haplotype analysis of high-impact variants within the candidate region can also be conducted to determine their association with phenotypes and to better understand the structure of variation and inheritance. Finally, the role of putative causative variants should be experimentally confirmed using reverse genetics approaches, such as in vitro assays, protein function assays, or gene editing techniques. These aspects warrant future investigation when adequate resources and animal samples become available, although they are beyond the scope of the present report.

## 4. Materials and Methods

### 4.1. Experimental Animals

This study involved 560 individuals (490 progeny from 30 sires and 40 dams) from a genetic improvement program aimed at enhancing disease resistance to Bacillary Necrosis of Pangasius (BNP) caused by *Edwardsiella ictaluri* in striped catfish at the Research Institute for Aquaculture No. 2 (RIA2), Vietnam [46]. Briefly, a nested mating design, in which one male was paired with two females, was practiced to produce 22 full- and 18 half-sib families (40 families). The fry from each family were raised in tanks (1.5 m^3^) for approximately three weeks until they reached an average body weight of 15–20 g, at which point they were physically tagged using Passive Integrated Transponder (PIT) tags.

The tagged fish were then acclimatized in cement tanks before being randomly assigned (approximately 32 individuals per family) to different cement tanks (10 m^3^) for a disease challenge test using a virulent *E. ictaluri* strain previously isolated at RIA2 [31]. Specifically, cohabitant fish (16.7 ± 6.1 g), initially injected with *E. ictaluri* at a dosage of 10^6^ CFU/0.2 mL per fish, were introduced into the cement tanks (10 m^3^) to be reared alongside the experimental fish at an approximate ratio of 1:3. After four days, additional bacteria were introduced into the tanks to maintain the bacterial density necessary for disease infection (10^5^ CFU per ml of rearing water). The experiment lasted for 23 days, during which survival data were recorded for this analysis.

### 4.2. Trait Measurements

During the 29-day challenge test, dead fish were collected and confirmed to have *E. ictaluri* infection through PCR and clinical symptoms [10]. These data included two main indicators of *E. ictaluri* resistance: survival status and survival time. Survival time was measured from the beginning of the test until the fish’s demise and was treated as a continuous trait, expressed in days. Survival status was treated as a binary trait (survived until the end of the experiment = 1; died = 0). Additionally, the weight of each fish at tagging was measured using a digital scale with a precision of 0.1 g.

### 4.3. Sequencing, Variant Calling and Genotype Data

A total of 560 fin tissue samples were sequenced using Diversity Arrays Technology sequencing (DArTseq™). Detailed information on DNA extraction and library construction is provided in Vu et al. [47]. Raw sequencing reads were processed through a series of filtering steps using Trim-Galore [48] to enhance sequence data quality: (i) trimming adapter sequences and contaminants, (ii) removing reads with >10% ambiguous bases (N), (iii) excluding all A-base reads, and (iv) eliminating low-quality and short reads (Q  ≤  20).

The filtered reads were then aligned to the reference genome (GCA_009078355.1) using the Burrows–Wheeler Aligner (BWA) [49] with the ‘mem’ algorithm, applying the parameters “-k 32 -M”. Subsequently, single-nucleotide polymorphisms (SNPs) were identified and underwent stringent quality control (QC) using bcftools-1.23 [50]. Only SNPs that passed these QC steps (Minor allele frequency > 0.05; quality score (QUAL) > 20, mapping quality (MQ) > 58) were considered accurate or classified as true positives. Additional QC ensured: (1) a minimum sequencing depth of 4× for both parents and offspring, (2) >90% of SNPs identified in each sample, with each SNP present in at least 90% of the samples, and (3) exclusion of SNPs displaying significant segregation distortion (*p* < 0.001) (Section 4.4 below). Polymorphic SNPs were identified by comparing the genotypes of the parental individuals. Missing data (about 10%) were then imputed and genotype data were phased using Beagle v5.5 [51].

### 4.4. Construction of Linkage Map

The linkage maps were constructed using the Lep-Map3 software, v.05 [52], which consists of three main steps.

Step 1: The VCF genotypes were converted to genotype likelihoods (posteriors) using the linkage2post.awk script provided with Lep-Map3. Then, missing parental genotypes were imputed using the ParentCall2 module, which accounts for half-sib families with the option outputParentPosteriors = 1, following the joining of parental genotype likelihoods for identical parents. The resultant dataset, including parental genotypes, was called without the outputParentPosteriors option and checked using the Filtering2 module to remove markers that exhibited significant segregation distortion (χ^2^ test, df = 1–2, *p* < 0.001, i.e., 1 in 1000 markers was removed by chance, separately for each family).

Step 2: SNP markers were assigned to linkage groups (LGs) using the SeparateChromosomes2 module. This module computed all pairwise LOD scores between markers and grouped markers accordingly. A LOD threshold of 10 and a minimum group size of 10 markers were used to define LGs, resulting in 30 groups consistent with the karyotype of striped catfish (2n = 60). Additional singleton markers were assigned to existing LGs using the JoinSingles2All module with LOD threshold of 8.

Step 3: Markers were ordered using the OrderMarkers2 module with the default parameters (useMorgan = 1, sexAveraged = 1). To verify the correctness of marker order within each LG, the LMPlot module was used to generate Lep-Map plots for each chromosome. Sex-specific map distances were calculated in centiMorgans (cM) using the Haldane mapping function (sexAveraged = 0). To estimate the average recombination rate across the genome, we applied a linear regression approach using sex-specific and sex-averaged genetic maps. For each of the 30 linkage groups (LGs), physical positions (in base pairs) were converted to megabases (Mb), and sex-specific genetic positions (in centiMorgans, cM) were extracted from the male and female maps. A sex-averaged genetic position was computed as the mean of the male and female cM values for each marker.

### 4.5. Synteny Analysis

We compared the genetic distance from a linkage map (in centiMorgans, cM) with the physical distance from an aforementioned reference genome (in base pairs, bp) (accession number GCF_009078355.1). These correlation analyses aimed to check if the reference genome order aligns with genetic linkage groups, or identify possible chromosomal rearrangements such as inversions, translocations, or mis-assemblies, as well as measuring variation in recombination across chromosomes.

### 4.6. QTL Mapping and Association Analysis

To identify QTLs linked to traits, we performed a genome scan using the R/qtl2 package, v0.38 [53]. Genotype, phenotype, covariate, and map information were formatted for R/qtl2 using a YAML configuration file. The population was treated as an F2-type cross, with multilocal genotypes, and additional covariates. Genetic and physical maps were provided in centimorgans and base pairs. Genotypes were encoded as A (homozygote reference), H (heterozygote), and B (homozygote alternative), and missing values were coded as “NA”, as required by the R/qtl2 package. The YAML configuration was imported into R/qtl2 using *read_cross2()* function, and pseudo markers were inserted at 1 cM intervals along each linkage group. Conditional genotype probabilities at all markers and pseudo markers were calculated assuming a genotyping error probability of 0.005. For each trait (survival status and survival time), we performed genome-wide QTL scans using a linear mixed model with a LOCO genomic relationship matrix. At a given locus *j*, the model can be written as

(1) $$y=\mu +X_{j}\beta_{j}+C_{\gamma }+u+e$$

where *y* is the vector of phenotypes (survival status and survival time), *μ* is the overall mean, *X*_*j*_ is the design matrix constructed from genotype probabilities at locus *j*, and *β*_*j*_ is the vector of additive QTL effects and *C* included the fixed effects of spawning batch, testing tank, and age (fitted as a continuous covariate), with corresponding coefficients *γ*. The random polygenic term captures background and follows the distribution as follows:

$$u∼\;N(0,\;\sigma_{u}^{2}K_{c(j)}^{LOCO})$$

where $K_{c(j)}^{LOCO}$ is the chromosomes-specific LOCO genomic relationship matrix calculated from all chromosomes except the one containing locus *j*. Residuals were assumed to follow

$$e\;∼\;N(0,\;I\sigma_{e}^{2})$$

At each genomic position, evidence for a QTL was summarized by the LOD score, defined as the log_10_ likelihood ratio comparing the full model including the locus-specific effect (*β* ≠ 0) with a reduced model without this effect. Genome-wide significance thresholds for each trait were obtained from 1000 permutation replicates using *scan1perm()*, applying the same LOCO kinship structure as in the genome scans. For comparison, we also evaluated a Haley–Knott model and a single-kinship LMM; however, the LOCO model was selected for final inference due to improved control of background polygenic effects.

The proportion of phenotypic variance explained by a significant QTL (PVE) was simply calculated as

$$PEV=({1-10}^{-2\times \frac{LOD}{n}})\times 100$$

where *n* is number of individuals and LOD is the LOD peak value. This formula is based on a widely used approximation that relates LOD scores to the coefficient of determination (R^2^), under the assumption of a linear model with normally distributed residuals [54]. Genotype–phenotype relationships at significant markers were visualized by plotting trait means (and their standard errors) for each genotype class.

### 4.7. Single-Step Genome-Wide Association Analyses (GWAS)

Single-step GWAS was performed using the same general model structure as in Equation (1), but implemented within the BLUPF90 family of programs [55]. The pedigree information was available to be tracked back to the base population, including 70 parents and 387 ancestors across 4 generations. GWAS running step by step were previously described [7,56]. The single-step approach combines pedigree and genomic information through a joint relationship matrix, allowing simultaneous estimation of breeding values for genotyped and non-genotyped animals. To enable direct comparison with the QTL results, we used the same SNP panel restricted to markers assigned to linkage groups. SNP effects were then derived from the single-step genomic evaluations and summarized in sliding genomic windows of 50 kb, defined based on the extent of linkage disequilibrium inferred from PopLDdecay [57] as described below.

### 4.8. Identification and Analysis of Candidate Genes

QTL analysis identified significant genomic regions associated with the studied traits. Candidate genes were subsequently identified based on the annotation and physical positions of SNP markers relative to the reference genome (Accession: GCF_009078355.1). Specifically, we performed linkage disequilibrium (LD) analysis using PopLDDecay [57], combined with 95% QTL confidence intervals, to narrow down the genomic regions where candidate genes are likely located on significant chromosomes. We then extracted the positions of these regions and extended both ends of each QTL flanking region for BLAST (v2.17.0) analysis. The extracted nucleotide sequences were searched against the GFF annotations of the striped catfish database (Accession: GCF_009078355.1) to identify candidate genes. Functional enrichment (GO, KEGG and COG) was performed in g:Profiler [58]. Significance was determined using the built-in g:SCS multiple-testing correction, and terms with adjusted *p* < 0.05 were considered enriched.

## 5. Conclusions

This study presents the first high-density SNP-based linkage map for striped catfish, constructed using a large multi-family pedigree and 8786 genome-anchored markers. The map revealed clear sex-specific differences in recombination rates and showed excellent concordance with the reference genome, providing a robust framework for downstream genomic analyses.

Using this map, we identified three significant QTLs on LG1, LG9 and LG29 associated with resistance to *Edwardsiella ictaluri*, the causative agent of Bacillary Necrosis of Pangasius (BNP). Although three QTLs explained a modest proportion of phenotypic variance, they represent the first genome-anchored locus associated with disease resistance in this species. Within the LD-defined interval surrounding the QTL, several biologically plausible candidate genes were identified, including *cd151*, *chid1*, *klf6*, *inhbaa*, and *slc15a5*, which have known roles in inflammatory and immune-regulatory pathways. Importantly, these genes should be viewed as prioritized candidates rather than functionally validated causal drivers, pending fine-mapping and functional evidence.

These findings substantially advance the genomic resources available for striped catfish and enhance our understanding of the genetic architecture underlying economically important traits. While further fine mapping, functional characterization and validation, and replication are required, the results provide a valuable foundation for future marker-assisted and genomic selection strategies aimed at improving disease resistance and overall performance in breeding programs.

## Figures and Tables

**Table 1:** Basic statistics and heritability (*h*^2^ ± se) for the traits studied.

Trait	Unit	*n*	Mean	SD	Range	*h* ^2^
Tag weight	Gram	490	20.8	11.7	7–77.9	0.74 ± 0.01
Time to death	Day	490	6.8	3.9	2–23	0.46 ± 0.09
Survival rate	%	490	32.2	46.8	0–100	0.44 ± 0.09

**Table 2:** Summary of quality control sequences mapped to a striped catfish assembly (including 30 chromosomes and 120 unplaced contigs = 732 Mb in length).

Sample	*n*	Avg. Cleaned Reads (Millions)	Mapped (%)	Average Reads Depth	Genome Breadth Coverage (%)	Genome Depth Coverage (×)
Female parent	40	2.29	90.2	22.9	0.755	0.18
Male parent	30	2.20	90.1	31.6	0.779	0.25
Offspring	490	1.61	88.7	19.6	0.685	0.14
All individuals	560	1.74	88.9	20.3	0.696	0.15

**Table 3:** Characteristics of 9882 filtered SNPs utilizing for Lep-MAP3.

Sample	*n*	Call Rate %	Minor Allele Frequency	FreqHets %	Discovered SNPs	QC SNPs
Female parent	40	99.0	0.428	31.9	336,782	9877
Male parent	30	99.0	0.433	33.0	336,782	9877
Offspring	490	98.5	0.426	31.5	336,782	9877
All individuals	560	98.5	0.426	31.7	336,782	9877

FreqHets: frequency of heterozygotes genotype (i.e., genotype coded 1 over summation of all three genotypes 0,1,2).

**Table 4:** Comparative linkage mapping statistics by linkage group: genome size, markers, sex-biased/sex-averaged map lengths, and recombination frequencies.

Linkage Group	Chromosome Length (Mb)	Number of Markers	Male-Biased Map Length (cM)	Female-Biased Map Length (cM)	Sex-Averaged Map Length (cM)	Average Inter-Marker Distance (cM)	Recombinant in Male (cM/Mb)	Recombinant in Female (cM/Mb)	Recombinant in Sex-Averaged (cM/Mb)
1	35.6	394	104.00	202.71	155.74	0.40	3.05	5.95	4.57
2	35.2	466	111.72	252.36	183.66	0.39	3.18	7.18	5.23
3	33.8	394	117.71	245.77	170.02	0.43	3.51	7.33	5.07
4	33.3	319	106.86	201.74	153.65	0.48	3.27	6.18	4.71
5	32.1	382	102.65	192.33	141.10	0.37	3.20	6.00	4.40
6	31.3	352	120.30	195.95	150.01	0.43	3.85	6.28	4.81
7	30.5	315	98.78	177.94	134.73	0.43	3.28	5.90	4.47
8	30.4	359	93.88	202.11	146.22	0.41	3.09	6.66	4.82
9	29.5	335	87.22	182.35	134.89	0.40	3.09	6.45	4.77
10	29.4	344	85.17	195.12	138.78	0.40	2.93	6.71	4.77
11	26.6	369	89.13	175.18	133.59	0.36	3.37	6.62	5.04
12	26.6	348	102.15	189.62	150.11	0.43	3.85	7.15	5.66
13	26.5	323	98.10	220.48	135.01	0.42	3.71	8.34	5.11
14	25.8	335	106.29	181.87	135.77	0.41	4.17	7.14	5.33
15	25.6	316	101.45	185.88	138.27	0.44	4.03	7.39	5.49
16	25.1	311	84.99	166.32	126.50	0.41	3.44	6.74	5.12
17	24.9	270	80.99	152.40	116.39	0.43	3.30	6.22	4.75
18	23.4	251	79.16	137.36	105.89	0.42	3.43	5.95	4.58
19	22.1	297	102.24	165.41	117.10	0.39	4.69	7.59	5.37
20	21.6	245	78.27	130.03	101.73	0.42	3.66	6.07	4.75
21	21.2	255	79.95	139.47	110.09	0.43	3.83	6.68	5.27
22	21.0	251	81.48	137.56	110.90	0.44	3.97	6.70	5.40
23	20.3	229	72.27	127.65	100.45	0.44	3.71	6.56	5.16
24	20.3	244	78.96	160.36	137.58	0.56	3.92	7.97	6.83
25	19.6	217	75.57	135.03	104.41	0.48	3.96	7.08	5.47
26	19.3	232	82.32	133.48	109.42	0.47	4.32	7.00	5.74
27	18.9	228	77.19	138.49	104.05	0.46	4.10	7.35	5.52
28	15.8	124	60.29	106.42	82.53	0.67	3.81	6.72	5.21
29	15.3	171	74.83	89.16	81.96	0.48	4.96	5.92	5.44
30	11.7	110	52.34	79.71	70.64	0.64	4.63	7.06	6.25
Sum	752.7	8786	2686.23	5000.24	3781.15	13.33	111.31	202.84	155.12
Mean	25.1	292.87	89.54	166.67	126.04	0.44	3.71	6.76	5.17
SD	6.2	80.71	16.20	41.45	26.23	0.07	0.51	0.62	0.52
Min	11.7	110.00	52.34	9.71	70.64	0.36	2.93	5.90	4.40
Max	35.6	466.00	120.30	252.36	183.66	0.67	4.96	8.34	6.83

**Table 5:** Additive (a) and dominance (d) effects for survival time at three significant QTLs.

Linkage Group	Peak Position (cM)	Additive Effect (a)	Dominance Effect (d)	% Change per Allele (a/Max × 100)	Genotype Means * (AA/AB/BB)
LG1	116.4	−0.56	−0.88	−6.1%	9.66/8.23/8.55
LG9	84.4	+2.62	+3.33	+28.7	6.49/12.43/11.72
LG29	54.2	−4.34	−4.59	−47.7	13.45/4.51/4.76

* computed based on survival time (day) of each genotype.

## Data Availability

The original contributions presented in this study are included in the article/Supplementary Materials. Further inquiries can be directed to the corresponding author(s).

## Supplementary Materials

The following supporting information can be downloaded at https://www.mdpi.com/article/10.3390/ijms27020784/s1.
