# Supplementary material for: First High-Density Linkage Map and Quantitative Trait Loci for Disease Resistance in Striped Catfish Pangasianodon hypophthalmus
Source: Int J Mol Sci. 2026 Jan 13;27(2):784. doi: 10.3390/ijms27020784 (PMC12841340; doi:10.3390/ijms27020784)
Supplement: Supplementary file 1 [file ijms-27-00784-s001.zip › ijms-4072433-supplementary.pdf]

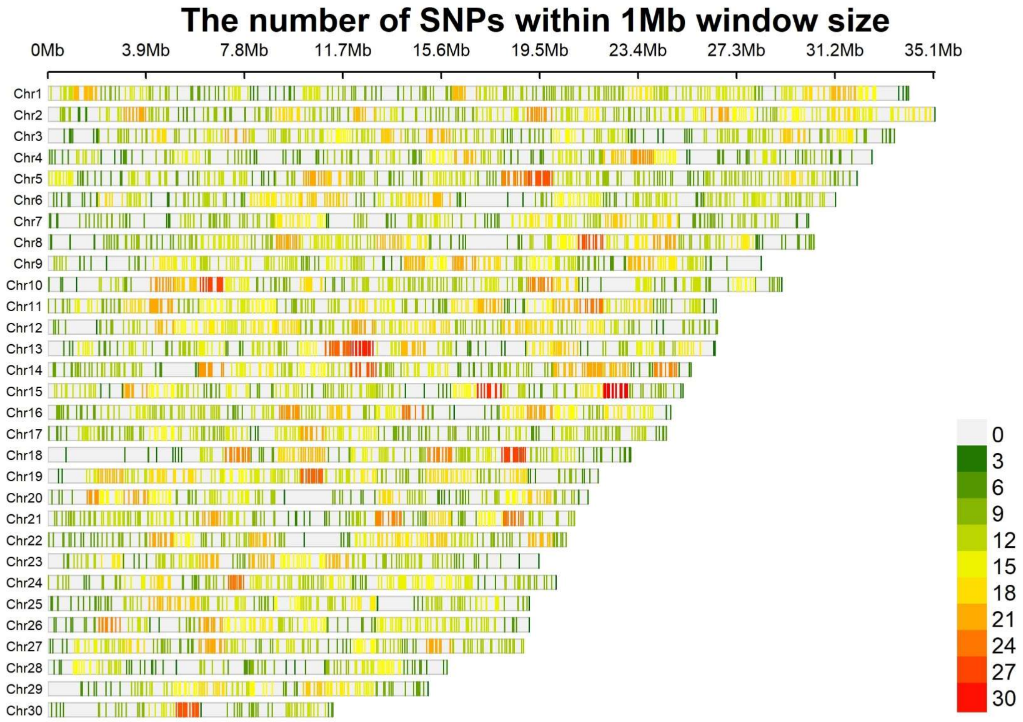

Supplementary Figure S1. SNP density across 30 chromosomes for 8,786 SNPs assigned to 30 linkage groups.

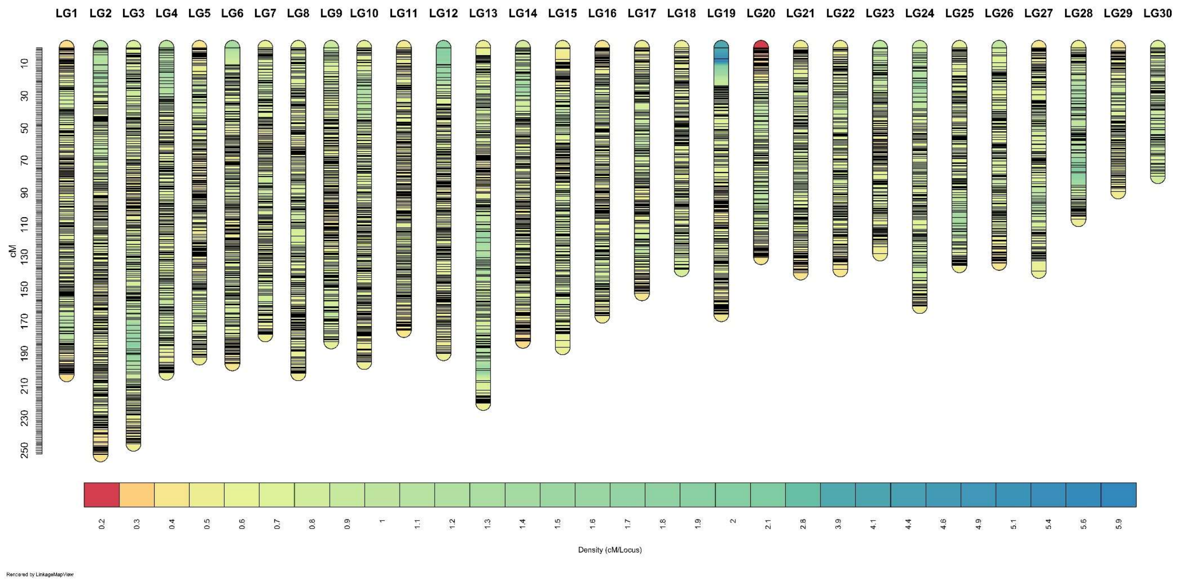

Supplementary Figure S2: Female-biased linkage map constructed using 8,786 markers assigning to 30 linkage groups.

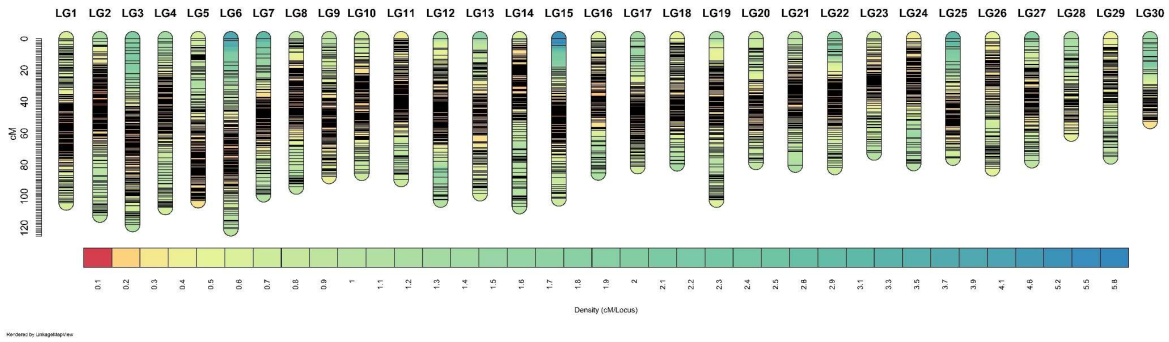

Supplementary Figure S3: Male-biased linkage map constructed using 8,786 markers assigning to 30 linkage groups.

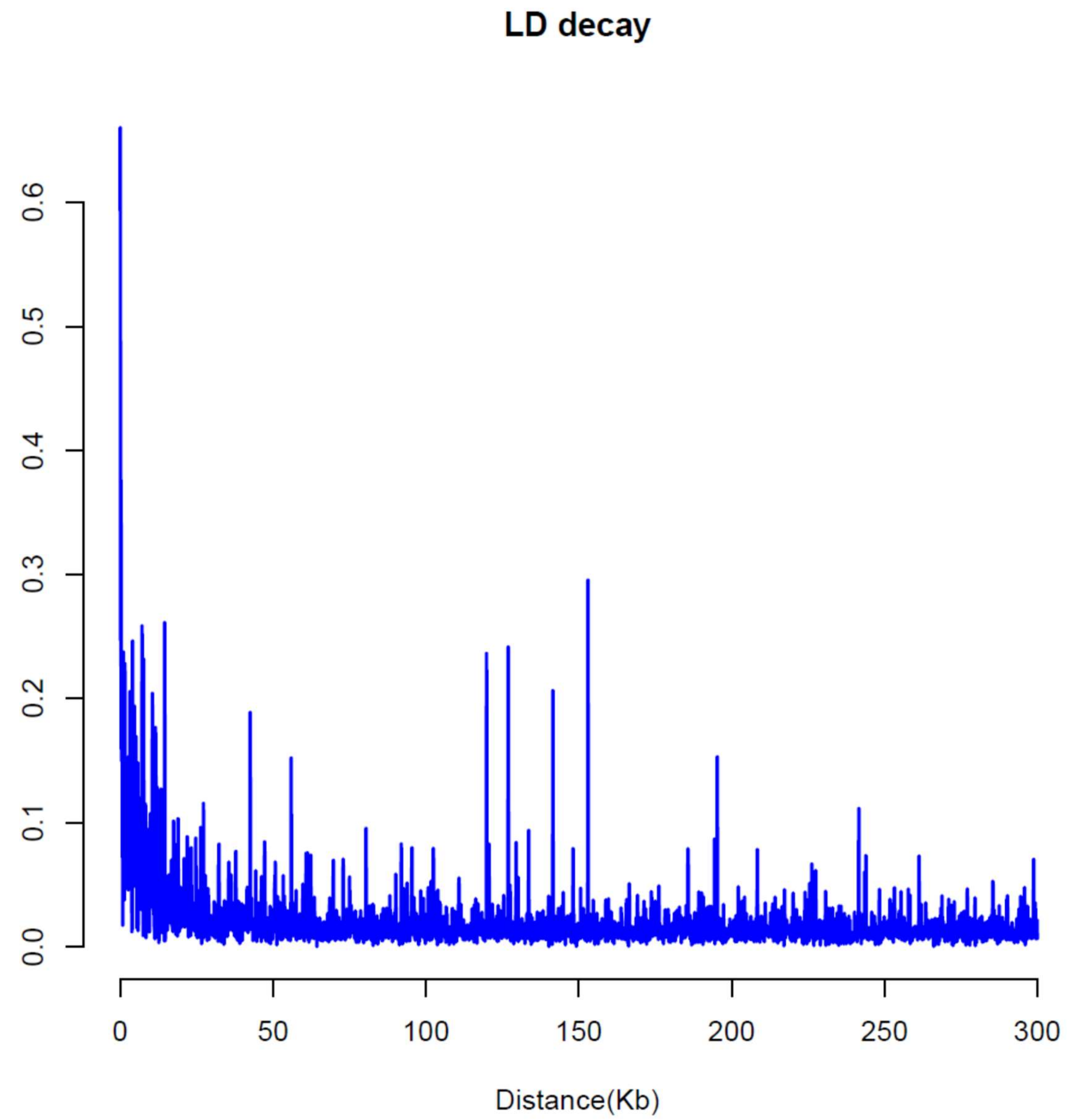

Supplementary Figure S4. Decay of linkage disequilibrium (r^2) with physical distance in the striped catfish breeding population. LD declines sharply within the first ~25 kb, supporting the use of a ±25 kb interval around the peak QTL for defining the candidate region.

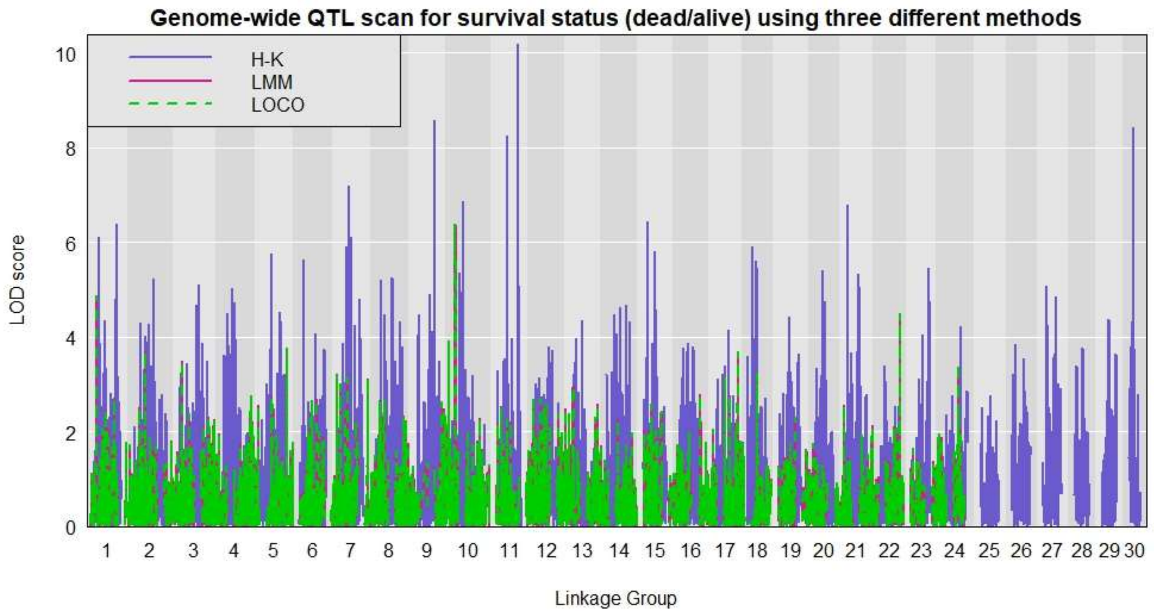

Supplementary Figure S5: Comparison of three QTL mapping approaches (Haley–Knott regression, single-kinship linear mixed model, and LOCO mixed model) for two disease-related traits. LOD profiles are shown for (left) survival status and (right) survival time across the 30 linkage groups. H–K produced inflated LOD scores due to unaccounted relatedness, whereas the single-kinship LMM partially reduced this inflation. The LOCO model showed the most conservative and stable behaviour, particularly near strong linkage signals, and was therefore selected as the primary analytical approach for all reported QTL results.

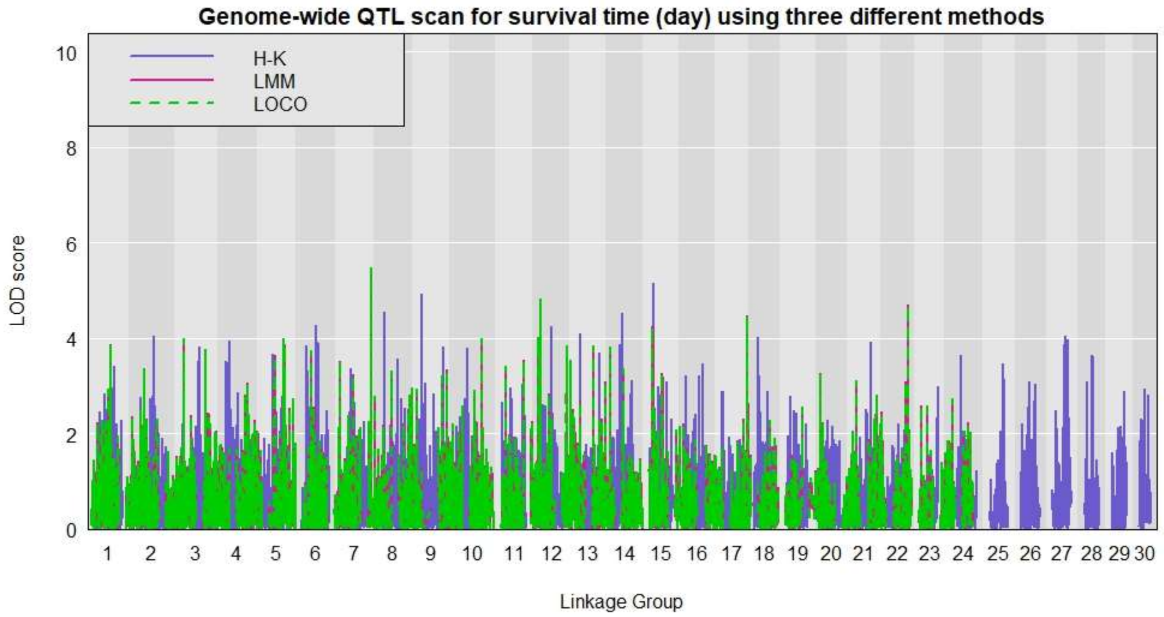

Supplementary Figure S6: Comparison of three QTL mapping approaches (Haley–Knott regression, single-kinship linear mixed model, and LOCO mixed model) for two disease-related traits. LOD profiles are shown for (left) survival status and (right) survival time across the 30 linkage groups. H–K produced inflated LOD scores due to unaccounted relatedness, whereas the single-kinship LMM partially reduced this inflation. The LOCO model showed the most conservative and stable behaviour, particularly near strong linkage signals, and was therefore selected as the primary analytical approach for all reported QTL results.

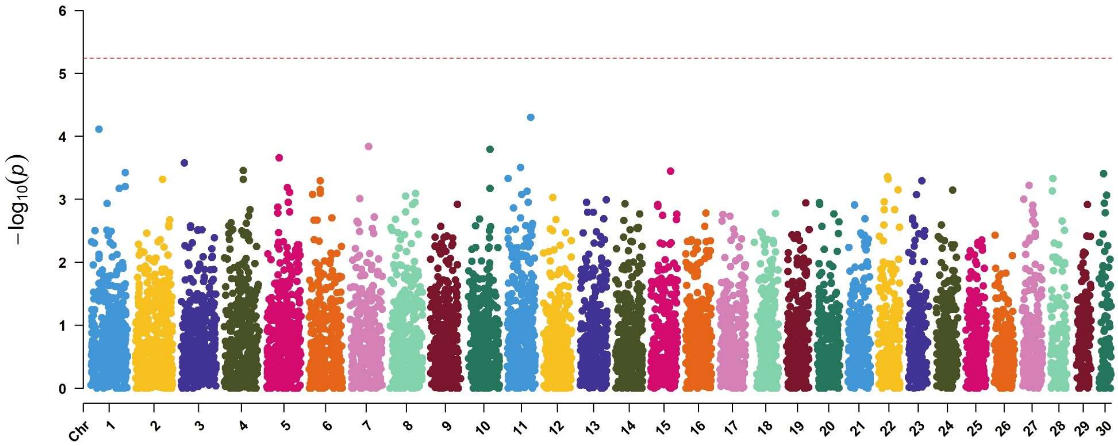

Supplementary Figure S7: Manhattan plot for GWAS across 8,786 SNPs across 30 chromosomes for disease resistance expressed as survival status (0=healthy, 1=dead).

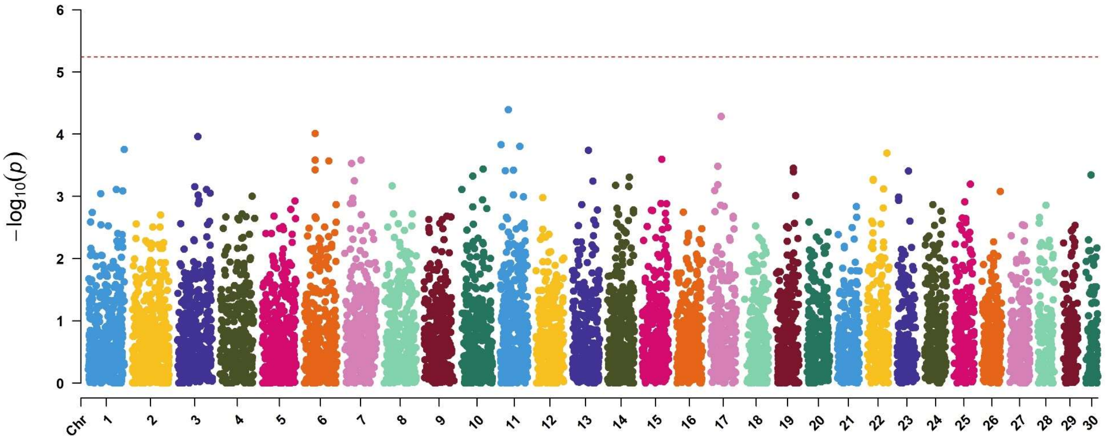

Supplementary Figure S8: Manhattan plot for GWAS across 8,786 SNPs across 30 chromosomes for disease resistance expressed as survival time (day to death

Supplementary Table S1. List of gene annotated when searching into striped catfish annotated database (Accession: GCF_009078355.1)

No	Feature count	Gene name	Description	Function	Disease resistance relevant
1	222	*tdrd12*	Tudor domain-containing protein 12	Germline development	No known immune function
2	195	*pfkpa*	Phosphofructokinase, muscle type	Glycolysis enzyme	Immune cells rely on glycolysis during activation; indirect
3	120	*pus7*	Pseudouridine synthase 7	RNA modification	Indirect link to stress responses
4	29	*ptprz1a*	Protein tyrosine phosphatase receptor type Z1	Neural signalling	Not known in fish immunity
5	28	*chid1*	Chitinase domain-containing protein	Chitin degradation, innate immunity	Chitinase-like proteins involved in microbial recognition and inflammatory responses
6	26	*top2b*	DNA topoisomerase II beta	DNA repair, transcription	Stress-response but not immune-specific
7	18	*cd151*	CD151 tetraspanin	Cell adhesion, membrane receptor	Tetraspanins modulate immune cell activation, pathogen entry, and viral infection
8	15	*urahb*	Urocanate hydratase B	Histidine metabolism	Not immune-related
9	13	*agbl2*	Alpha-1,4-glucosyltransferase B	Glycogen metabolism	Not known in fish immunity
10	11	*slc15a5*	Solute carrier family 15	Peptide/H^+ transporter	Some SLC15 members transport microbial peptides → innate immunity
11	11	*tmem17*	Transmembrane protein 17	Ciliary signalling	No known immunity link
12	8	*gatd1*	Glutamine amidase-related protein	Amino acid metabolism	Not known
13	5	*klf6a*	Kruppel-like factor 6	Transcription factor	KLF family regulates macrophage activation
14	4	*triqk*	Triple QxxK protein	Unknown	Unknown
15	3	*inhbaa*	Inhibin beta A	Growth factor, TGF-β family	TGF-β pathway modulates inflammation and immunity
16	3	*srpk2*	Serine/arginine protein kinase 2	RNA splicing regulation	Indirect role; SRPK family modulates antiviral response
17	2	*fzd8a*	Frizzled-8 receptor	Wnt signalling receptor	Wnt pathway regulates immune cell differentiation

Supplementary Table S2. Detailed enrichment statistics for genes within the QTL regions.

GO Term ID	Term name	Category	p-value	Intersection Size	Genes contributing
**GO:0030097**	Hemopoiesis	GO:BP	0.0261	6	*cd151, chid1, inhbaa, klf6a, slc15a5, srpk2*
**GO:0002244**	Hematopoietic progenitor cell differentiation	GO:BP	0.0277	3	*cd151, klf6a, inhbaa*
**GO:0030098**	Lymphocyte differentiation	GO:BP	0.0277	4	*cd151, chid1, klf6a, inhbaa*
**GO:0030183**	B cell differentiation	GO:BP	0.0277	3	*cd151, klf6a, inhbaa*
**GO:0045070**	Positive regulation of viral genome replication	GO:BP	0.0277	2	*inhbaa, srpk2*
**GO:0046649**	Lymphocyte activation	GO:BP	0.0277	5	*cd151, chid1, klf6a, inhbaa, slc15a5*
**GO:0045321**	Leukocyte activation	GO:BP	0.0427	5	*cd151, chid1, klf6a, inhbaa, slc15a5*
**GO:1903131**	Mononuclear cell differentiation	GO:BP	0.0427	4	*cd151, klf6a, inhbaa, chid1*
**GO:0005945**	6-phosphofructokinase complex	GO:CC	0.0372	1	*pfkpa*
**GO:0043509**	Activin A complex	GO:CC	0.0372	1	*inhbaa*
**GO:0043511**	Inhibin complex	GO:CC	0.0372	1	*inhbaa*
**GO:0043512**	Inhibin A complex	GO:CC	0.0372	1	*inhbaa*
**GO:0048180**	Activin complex	GO:CC	0.0372	1	*inhbaa*
**GO:1990923**	PET complex	GO:CC	0.0372	1	*pfkpa*
**GO:1990851**	Wnt–Frizzled–LRP5/6 complex	GO:CC	0.0425	1	*fzd8a*
